# Unsupervised Temporal Contiguity Experience Does Not Break the Invariance of Orientation Selectivity Across Spatial Frequency

**DOI:** 10.3389/fnsys.2019.00022

**Published:** 2019-05-28

**Authors:** Els Crijns, Dzmitry A. Kaliukhovich, Lara Vankelecom, Hans Op de Beeck

**Affiliations:** ^1^Laboratory of Biological Psychology, Department of Brain and Cognition, KU Leuven, Leuven, Belgium; ^2^Leuven Brain Institute, Leuven, Belgium

**Keywords:** V1, orientation selectivity, temporal contiguity hypothesis, spatial frequency, rodents

## Abstract

The images projected onto the retina can vary widely for a single object. Despite these transformations primates can quickly and reliably recognize objects. At the neural level, transformation tolerance in monkey inferotemporal cortex is affected by the temporal contiguity statistics of the visual input. Here we investigated whether temporal contiguity learning also influences the basic feature detectors in lower levels of the visual hierarchy, in particular the independent coding of orientation and spatial frequency (SF) in primary visual cortex. Eight male Long Evans rats were repeatedly exposed to a temporal transition between two gratings that changed in SF and had either the same (control SF) or a different (swap SF) orientation. Electrophysiological evidence showed that the responses of single neurons during this exposure were sensitive to the change in orientation. Nevertheless, the tolerance of orientation selectivity for changes in SF was unaffected by the temporal contiguity manipulation, as observed in 239 single neurons isolated pre-exposure and 234 post-exposure. Temporal contiguity learning did not affect orientation selectivity in V1. The basic filter mechanisms that characterize V1 processing seem unaffected by temporal contiguity manipulations.

## Introduction

The mammalian visual system is organized hierarchically. Hubel and Wiesel (1959) initially described the properties in the first stage of this cortical system, the primary visual cortex (V1), with neurons responding to a specific orientation and/or direction. Since these first observations in cats, these coding principles have been described in many mammals, including rodents (Girman et al., 1999; Niell and Stryker, 2008; Glickfeld et al., 2013; Kaschube, 2014; Li et al., 2015; Scholl et al., 2017). A second emerging property of V1 is the tuning of neurons to an optimal spatial frequency (SF) (Girman et al., 1999) which is largely independent from orientation preference (Webster and De Valois, 1985; Mazer et al., 2002). The selectivity for these dimensions is to some degree experience dependent (Kreile et al., 2011). V1 is classically seen as a static feature detector, however, experience-dependent plasticity and adaptation have been observed in V1 (Gilbert and Li, 2012). Over-exposing juvenile rats for several weeks to a specific orientation leads to overrepresentation of that orientation in the V1 (O’Hashi et al., 2007). Although the outcome and mechanisms might differ, plasticity of orientation tuning have been observed in adult mice (Yoshida et al., 2012) after over-exposing them to a specific orientation. In cats this over-exposure causes a repulsive shift in orientation preference of V1 neurons (Dragoi et al., 2000). A shift in the orientation tuning curve can also be induced by short term exposure to a grating with the optimal orientation followed by a grating with a 15° orientation difference (Yao and Dan, 2001). Despite the evidence that the tuning along an individual dimension can be influenced by exposure, the concept that the two dimensions are coded more or less independently has not been challenged through learning paradigms (Mazer et al., 2002; Gao et al., 2010).

Tuning properties change markedly in higher levels of the visual system. In the highest level, in monkeys known as the inferior temporal cortex (ITC), neurons show a tuning for complex object properties (Tanaka, 1996). In addition, this tuning is relatively invariant for transformations, such as changes in object size and position (Rolls and Stringer, 2006; Afraz et al., 2014). It has been proposed that such tuning properties are acquired through experience and exposure to the spatiotemporal statistics of the environment. One specific hypothesis is temporal contiguity learning. According to this hypothesis the cortex makes use of the natural tendency for objects to be presented as a series of temporally contiguous retinal images, each with slightly different properties (Li and DiCarlo, 2008). Combining these images will lead to building neural object representations that are invariant to identity-preserving transformations.

The validity of this hypothesis has been tested in primates by exposing subjects to an altered visual experience. In one experiment multi-unit extracellular recordings were performed in monkey ITC (Li and DiCarlo, 2010). A medium sized preferred object, shown to reliably induce a response in that neuronal site, was presented at the center of gaze. After 100 ms the object changed in size. If the change was to the ‘swap’ size (e.g., the object becomes larger) the object identity would change toward a non-preferred object. If the change was toward the ‘control’ size (e.g., the object becomes smaller) the object identity did not change. After repeated exposure the neural responses would gradually become more responsive to the non-preferred object, but only at the swap size, as predicted by the temporal contiguity hypothesis. Similar effects had been shown previously for position invariance in monkey ITC (Li and DiCarlo, 2008). The change in selectivity was rapid, detectable after only 100 exposures over 15 minutes, and even leading to a reversal of selectivity after ∼2 h. Converging behavioral evidence for temporal contiguity learning has been obtained in humans for position invariance of objects (Cox et al., 2005) and for rotation invariance of faces (Wallis and Rolls, 1997). An fMRI adaptation experiment in humans also suggested an influence of temporal contiguity learning on view-point invariance of faces (Van Meel and Op de Beeck, 2018). However, the observed behavioral effects were much smaller than the electrophysiological results discussed above. The mechanism and source of this phenomenon remains unclear. Yet, the time course is consistent with synaptic plasticity, and computational-models have been proposed that mimic this learning mechanism using Hebbian-like learning rules (Wallis and Rolls, 1997; Wiskott and Sejnowski, 2002; Sprekeler et al., 2007).

Although large effects have been described when manipulating temporal contiguity learning, there are only a limited amount of studies investigating these effects. Little is known about the extent of brain regions involved, or to which stimulus properties it applies. More specifically, it is unclear to what extent temporal contiguity learning affects tuning properties also at lower levels in the visual hierarchy. Models of object recognition would often assume that object representations are malleable to experience, but that the basic filter characteristics in areas such as primary visual cortex are more stable. Therefore we investigated whether temporal contiguity manipulations could affect the well-established independent coding of orientation and SF in primary visual cortex (Mazer et al., 2002). Additionally, we examined the responses during the exposure phase to investigate how neurons process the different stimulus pairs and verify that neurons are sensitive to the temporal contiguity manipulation during exposure.

We modeled our study in line with the aforementioned experiment on changing the tolerance of object selectivity for transformations in size in monkey IT (Li and DiCarlo, 2010). In our case, we studied the effect of temporal contiguity learning in the primary visual cortex of rats, challenging the tolerance of orientation selectivity for transformations in SF. During training, a grating of the preferred orientation was presented using a medium SF. After 200 ms the grating changed in SF. If the change was to the ‘swap’ SF (e.g., the grating decreases in SF) the grating orientation would change toward a non-preferred orientation. If the change was toward the ‘control’ SF (e.g., the grating increases in SF) the grating orientation did not change. Based on the findings of Li and DiCarlo (2010), we expected the selectivity to decrease at the swap SF if temporal contiguity would also play a role for feature coding in V1. To test this, we performed extracellular recordings in rat V1. The orientation selectivity of isolated single-units was determined before and after exposure to the altered visual experience. Contrary to the temporal contiguity hypothesis the orientation selectivity remained the same in the swap and the control SF. Our study further expands the knowledge on the extent and limitations of temporal contiguity learning.

## Materials and Methods

Experiments were performed in eight male Long Evans rats (Janvier Labs, Le Genest-Saint-Isle, France) aged 10–32 weeks at time of surgery. Animals were individually housed upon arrival. The protocol was approved by the Ethical Committee for Animal Experiments at the KU Leuven (P119/2014).

### Surgery

**FIGURE 1:**
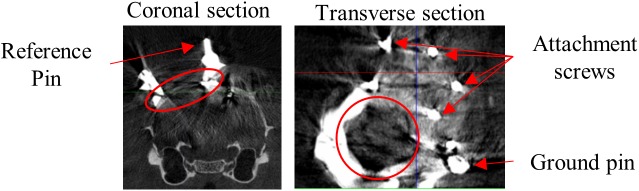
CT scan. Representative example CT scan sections of one animal. Coronal section shows medial reference wire imbedded in dental cement which surrounds the craniotomy. Transverse section shows part of the recording chamber surrounding the craniotomy, the pin for attachment of the probe ground wire and screws for attachment of the denture acrylic. Red circles encompass the craniotomy.

Surgery procedures and materials were similar to Kaliukhovich and Op de Beeck (2018). We will briefly review the procedures, and in particular mention the differences. Rats were anesthetized using isoflurane (1.5–3% at 1 l/min O_2_) and placed in a stereotaxic frame (Neurostar, Kopf Instruments, Tujunga, CA, United States). Eyes were covered with white Vaseline (Qualiphar, Belgium) to prevent corneal damage. Lidocaine (2%, 0.1 ml SC) was administered for local analgesia. The skin and connective tissue were removed and the skull was thoroughly cleaned. A head-post was attached to the frontal bone with UV-cure dental cement (Tetric Evoflow, Ivoclar Vivadent, Schaan, Liechtenstein). For improved adhesion of the denture acrylic, screws were implanted around the craniotomy (Figure 1).

A craniotomy (Ø = ∼2 mm) was made above the left visual cortex (Espinoza and Thomas, 1983) medial to the lateral ridge, and anterior to the posterior ridge. This includes V1 target positions described earlier (Vermaercke et al., 2014; Vinken et al., 2014, 2017; Kaliukhovich and Op de Beeck, 2018). A recording chamber surrounding the craniotomy was built from UV-cure dental cement, including three metal reference wires and a ground pin. The craniotomy was sealed with silicon (Kwik-Cast, WPI, Sarasota, FL, United States). Finally, the remainder of the exposed skull was covered with denture acrylic (Paladur, Heraeus Kulzer International, Hanau, Germany) to provide additional stability. Each surgery lasted approximately 2.5 h and animals were allowed to spontaneously recover in their home cage.

After recovery a CT-scan (SkyScan 1076 *in vivo* micro-CT; Bruker Co., Billerica, MA, United States) was made to confirm the position of the craniotomy above V1 and define the coordinates of the reference wires with respect to bregma.

### Electrophysiological Recordings

After at least 1 week of recovery, habituation to the recording set-up was performed as previously described (Vermaercke et al., 2014; Vinken et al., 2017). Rats received ∼12 ml water per day immediately after training or recording for the entire duration of experiments. The body weight was monitored to remain above 85% of pre-training weight. The habituation to head fixation happened gradually, with an increased duration of daily sessions from 30 s to 1 h 30. The head-post was fixed in a metal arm connected to a stereotaxic frame (Kopf Instruments, Tujunga, CA, United States). The animal’s body was covered by a wooden box to provide cover and limit movement.

Apparatus and recording procedures were previously described (Vermaercke et al., 2014; Kaliukhovich and Op de Beeck, 2018). Rats were placed at a distance of 30 cm from a 24-inch LCD monitor (resolution = 1280 pixels × 768 pixels). Stimulus onset was detected through a photocell at the bottom right corner of the screen and synchronized with the neural signal through the Neuralynx Digital Lynx system and Cheetah software (Neuralynx, Bozeman, MT, United States). We used 32-channel linear probes (A1x32-Edge-10mm-20-177-CM32, NeuroNexus, Ann Arbor, MI, United States). The position of the probe was manipulated with a stereotaxic arm and the recording locations were determined relative to the reference wires around the craniotomy. The probe location relative to bregma can then be determined based on the CT-scans (Figure 1). The probe ground wire was attached to the ground pin imbedded in the dental cement, which penetrated the saline-filled recording chamber. The upper channel of the probe was used as an internal reference for recordings.

The probe was lowered orthogonally into the cortex until spiking activity was observed. After the brain tissue was stabilized, a receptive field (RF) mapping test was performed and peri-stimulus time histograms (PSTHs) were plotted online (Kaliukhovich and Op de Beeck, 2018). The RF mapping test consisted of a randomized repetition of 23 non-overlapping flashing white squares (213 pixels × 196 pixels, 8.0 cm × 8.66 cm, 14.2° × 16.8°) of 300 ms each, on a black background. The RF positions were compared between recording locations to identify the retinotopic map as described for V1 (Espinoza and Thomas, 1983; Vermaercke et al., 2014). Moving the probe between penetrations along the antero-posterior, and latero-medial axis should cause a predictable shift in RF. If stable visual responses were present at several RF positions the selectivity test (see below) was performed. The RF mapping was only used to define the localization of the probe in V1, not to adjust stimulus characteristics in any way.

**FIGURE 2:**
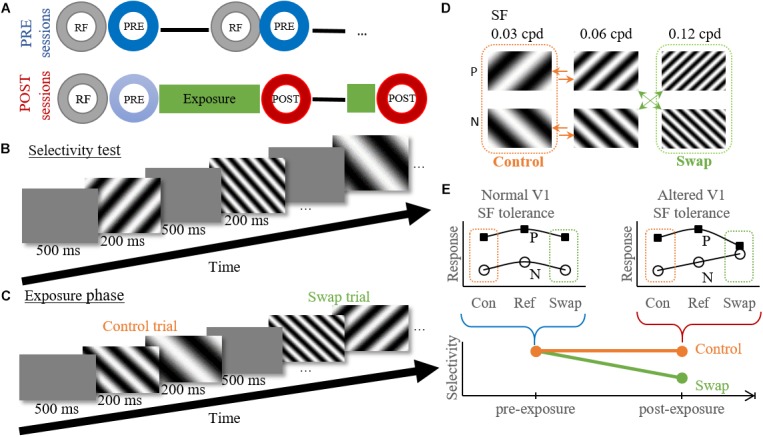
Experimental design and temporal contiguity hypothesis. **(A)** Within session design. Circles indicate different experimental tests. RF, RF mapping; PRE/POST, Selectivity test pre- or post-exposure. **—** electrode movement to new position (PRE) or depth (POST). **(B)** Example time course of the selectivity test. **(C)** Example time course of the exposure phase for the high swap group. Control trial shows the transition from reference to low SF, without a change in orientation. Swap trial shows a transition from high to reference SF with the associated change in orientation. **(D)** Exposure protocol for the high swap group. We show the six gratings used for both the selectivity test and exposure phase, with P the preferred orientation and N the non-preferred orientation for individual neurons. The green (swap) and orange (control) arrows indicate the eight possible stimulus combinations during the exposure phase. The order of the stimulus pairs is randomized and counterbalanced within each session. Note that the assignment of low and high SF to swap and control condition is counterbalanced between animals. **(E)** Graphical explanation of the hypothesis. Pre-exposure the orientation selectivity is tolerant to SF changes as seen by the parallel tuning curves for both orientations. According to the temporal contiguity hypothesis the tolerance would decrease post-exposure for the swap SF, but not for the control SF. A decreased selectivity would thus be observed for the swap SF but not for the control SF. Con, control SF; Ref, reference SF; Swap, Swap SF.

During pre-exposure sessions (Figure 2A) this selectivity test was repeated at several cortical locations for maximum 3 h or until the animal showed signs of stress. If it could be confirmed that a probe location was situated in V1 online and offline (see below), the data collected during these selectivity tests was grouped as pre-exposure data. This data only included data recorded before the first exposure session of that animal. Note that we often penetrated at several locations during one pre-exposure recording session (Figure 2A), and performed at least two sessions per animal.

For post-exposure sessions (Figure 2A) the RF mapping and selectivity test were followed by the exposure phase and a post-selectivity test, with possible a second, shorter, exposure phase and post-selectivity test at a different recording depth. As of the second post-exposure session the initial selectivity test (indicated in light blue on Figure 2A) was not evaluated since these cells were no longer naïve. It is unknown how the exposure of previous days would affect neural selectivity on the present day. This means that only selectivity data collected immediately after an exposure phase was grouped as post-exposure data, and pre-exposure data included all data collected before the first exposure phase. We only penetrated at one location in each post-exposure recording session, and we aimed to target similar cortical locations as in the pre-exposure sessions. The overall match can be qualitatively evaluated through a comparison of the population receptive fields (Figure 4B).

#### Stimuli

We set out to challenge the independent coding of orientation and SF in V1. Working with these stimulus features limited our degrees of freedom in stimulus design. There is only a finite number of SFs and orientations that can be used, while defining distinct stimuli. Therefore, rather than optimizing the stimulus to each recording location, we selected a set of six stimuli that would be able to drive a wide range of neurons. Diagonal orientations (45 and 135°) were chosen to circumvent the bias toward the horizontal orientation. A 90° separation between the two orientations is the maximum difference that can be defined. Since most neurons have an orientation tuning band width of 90° or less (Girman et al., 1999), this should be sufficient to differentiate between the two orientations. If any neurons were responsive to both orientations to the same degree, it would have been excluded by our selection criteria, since it will not show selectivity (see below). Most neurons show an optimal response at 0.08 cycles per degree (cpd) and a cut off SF above 0.15 cpd (Girman et al., 1999). Our selection of three SFs at 0.03, 0.06, and 0.12 cpd should thus be able to drive most neurons in V1. Also the use of multi-channel probes favors the use of default stimuli rather than stimuli tailored to individual neurons, since multiple neurons will be recorded at each probe location.

Finally, since we do not know how the exposure of previous days would affect later recordings, stimuli were presented full screen (52 cm × 32 cm or 81.83° × 56.15°), both during recording and passive exposure. Rather than limiting the exposure to the receptive field of individual neurons, we covered as much of the visual field as possible, so all V1 neurons received the same amount of exposure. This allowed us to group data across multiple positions and days.

#### Selectivity Test

During the selectivity test six full-screen gratings, combining the two orientations and three SFs, were presented in a random order for minimally 60 (median = 65) presentations each. Each stimulus presentation lasted 200 ms and was followed by a blank inter-stimulus interval of 500 ms (Figure 2B,D).

#### Exposure Phase

The exposure phase was intended to expose the rats to an altered temporal contiguity (Figure 2C,D) trying to reduce the SF tolerance of orientation selectivity in V1, as was done before for the size tolerance of shape selectivity in monkey ITC (Li and DiCarlo, 2010). The same six gratings were used as during the selectivity test, grouped per two. The exposure phase consisted of eight unique stimulus pairs. Stimulus pairs were shown in random order. Each pair contained the reference SF (0.06 cpd) combined with the high or low SF, in random presentation order (arrows in Figure 2D). Each grating was shown for 200 ms without an interval between the two stimuli of the pair (Figure 2C). There was a 500 ms blank interval between pairs. During the first exposure phase in a recording location there were 400 exposure trials per stimulus pair, lasting 48 min (8^∗^400^∗^[0.2 + 0.2 + 0.5] sec). For most positions a second exposure phase was performed at a new depth, which lasted only 12 min and contained 100 trials per stimulus pair.

The eight stimulus pairs can be divided into two experimental conditions (Figure 2D). In the control condition both stimuli had the same orientation (45° or 135°). In the swap condition, each animal either experienced a change in orientation at the high SF (three animals) or at the low SF (five animals). In a single animal, the exposure was the same across exposure phases and across sessions. As a consequence, the effect of the exposure could possibly build up across sessions in addition to the within-session effects reported before (Li and DiCarlo, 2008, 2010).

### Experimental Design and Statistical Analysis

Once data were collected, spike detection and clustering were performed in six partially overlapping groups or ‘chunks’ of seven probe channels. For spike detection a 500 Hz high-pass filter was applied. Next, spikes were identified based on a double-threshold algorithm (SpikeDetect, Kadir et al., 2014). Only when the signal exceeded three standard deviations above noise, it was considered a spike. All connected components (in time or space) crossing the two standard deviation threshold were considered part of the same spike. Finally, the waveform was extracted and principle component analysis was performed extracting five features. A masked EM algorithm was used for clustering analysis (KlustaKwik, Kadir et al., 2014). The number of clusters was defined automatically, with a maximum of 30, after a series of splitting and merging events based on the feature and mask vectors of each spike. This yielded a total of 887 clusters across 44 sessions pre-exposure, and 877 clusters across 34 sessions post-exposure (Table 1).

**Table 1:** Overview of obtained data per condition and per phase.

		Pre-exposure			Post-exposure		
	Rats	Sessions	Clusters	SUs	Sessions	Clusters	SUs
Low	5	26	486	112	23	603	154
High	3	18	401	127	11	274	80
**TOTAL**	**8**	**44**	**887**	**239**	**34**	**877**	**234**


*The table indicates the numbers of rats used per condition. The number of sessions performed across rats, the clusters isolated form these sessions and the amount of selective SUs are shown per condition (rows) and per phase (columns).*

Finally there was a manual verification of the clusters with KlustaViewa (Rossant et al., 2016). Based on the principle component analysis, spike waveforms and inter-spike interval histograms of individual clusters, we identified putative single units (SUs) and excluded noise and Multi-Unit (MU) clusters.

#### RF Mapping Data

Receptive field mapping data were collected at the beginning of each recording session. We did not identify SUs for the RF mapping data, because the time interval between RF mapping and the selectivity tests was often too long to guarantee finding the same SUs. Thus, we combined all data from one recording location into one large MU cluster, only including those locations in which SUs were found in the selectivity test (see section “Selectivity Data”). All further calculations of the RF mapping data were applied to these MU clusters. The main goal of this RF mapping analysis was to determine that the recorded MU population was visually responsive to some positions on the screen, to identify the retinotopic map, and to compare the overall RF positions of pre- and post-sessions. It was not used to adapt the stimuli in any way to the RF properties, because all shown stimuli were full-screen gratings using a pre-defined set of orientations and SFs.

We calculated mean MU responses for the time interval from 15 to 115 ms after stimulus onset. We also identified the spontaneous firing rate (baseline, BL) in a time interval from 95 ms before stimulus onset to 5 ms after. MU responses were averaged over trials per screen position. All positions with a response significantly different from baseline were considered as part of the MU RF (one-sided *t*-test from BL, *p* < 0.05). RF positions are plotted as a heat map representing the amount of times a specific position was significant over all locations (Figure 4B). The heat maps are compared pre- and post-exposure to show that both sets of SUs were collected from a similar neural population.

#### Exposure Data

Response suppression with repeated exposure is well described, but has not been investigated in relation to temporal contiguity learning. We investigated whether there was a difference in response adaptation between the two exposure conditions of swap and control trials. Only the recording from the first 50 trials per exposure condition were collected per session. Data was available for 30 exposure sessions across animals. As for the RF mapping data, exposure data was combined for all probe channels to form one large MU cluster. From this MU data, a PSTH was calculated for every trial from 50 ms before stimulus onset until 50 ms after second stimulus offset. For each stimulus pair these PSTHs were averaged over trials and normalized by dividing them by the peak value of the stimulus pair with the largest peak value. These normalized PSTHs were then grouped and averaged separately for control and swap conditions per session. The difference in peak response between the first and second stimulus of each condition was investigated, as well as the difference between conditions for each stimulus of the pair (paired *t*-test). The difference in response to the second stimulus was further analyzed by pairwise comparisons between conditions for each time bin (paired *t*-test with Bonferroni correction).

#### Selectivity Data

**FIGURE 3:**
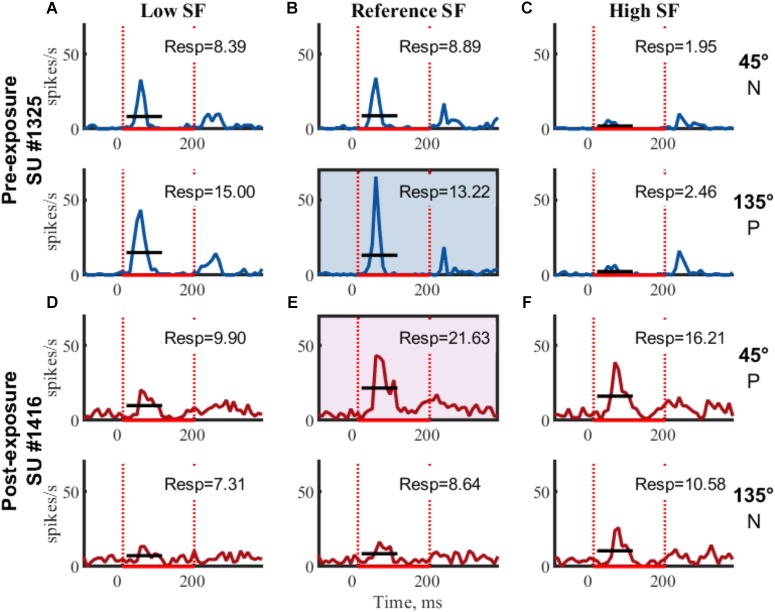
Example SUs. PSTHs of raw FR (mean across trials) for two example SUs are shown for each of the six gratings. Rows indicate same orientation (P, preferred orientation; N, non-preferred orientation; determined *post hoc*), columns indicate same SF. Boxed PSTHs indicate reference stimulus for normalization (see Table 2). Resp = average FR from 15 to 115 ms in spikes/s; 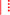 stimulus presentation; **—** mean response FR.

Further selection criteria were applied to each of the clusters identified as putative single units. A single PSTH was generated per stimulus (see Figure 3 for example SUs). Baseline responses were calculated over a 100 ms time window, starting 95 ms before stimulus onset, across all stimulus conditions. Clusters with an outlier baseline response above 50 spikes/sec (only 2 clusters out of the final 475 SUs) were excluded because these clusters are unlikely to be SUs [see Mruczek and Sheinberg (2012) for example distributions of SU spontaneous firing rates]. Next, the baseline response was subtracted from the PSTHs and the average stimulus response (FR, see Table 2 for example SUs) was calculated from 15 to 115 ms after stimulus onset. For inclusion in the dataset, SUs had to be significantly responsive (one-sided *t*-test from 0, *p* < 0.05, ^∗^ on Table 2) to at least one stimulus.

**Table 2:** Example SUs as shown in Figure 3.

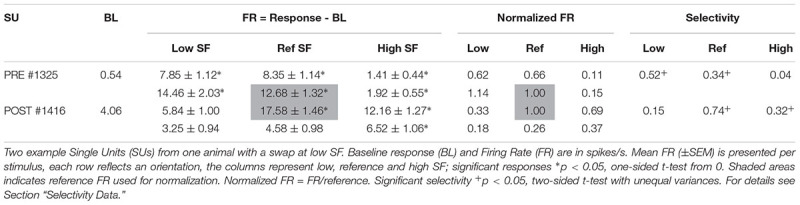

The orientation with the highest response at the reference SF was considered the preferred orientation. Stimulus responses were normalized by dividing them with the response to the preferred orientation at the reference SF (Figure 3 and Table 2, FR in shaded area). The amount of orientation selectivity was defined as the difference in average normalized response between the preferred and non-preferred orientation at each SF (Table 2, Selectivity).

Finally, selective SUs were identified based on a significant difference (two-sample *t*-test with unequal variances, *p* < 0.05) between the trial-based responses (15–115 ms) to the two orientations in the reference SF. Unless otherwise noted, analyses are performed on these selective putative SUs.

To assess the pre-exposure selectivity (PRE), all SUs from sessions before the first exposure were grouped. Post-exposure selectivity (POST) combines SUs from the selectivity tests right after each exposure phase. We expected a reduced orientation selectivity at the swapped SF after exposure, whereas the selectivity at the control SF should not be altered.

To answer this question, we tried to balance the number of SUs between pre- and post-exposure, irrespective of swap condition (Table 1). Additionally, whether the swap condition involved the high or the low SF was balanced across animals so that the amount of selective single units pre-exposure was approximately equal between swap groups. Due to the small number of pre-exposure SUs in two of the low swap animals (Figure 7, #577: *n* = 12 and #435: *n* = 5) there was an imbalance between swap conditions in post-exposure SUs and in the number of animals (Table 1).

The pre- and post-exposure SUs were assessed for significant selectivity at each SF (*t*-test from 0) and between SFs (paired *t*-tests) on a group level. To assess our main hypothesis of an altered selectivity post-exposure at the swap SF, but not at the control SF, an unpaired t-test was performed for both the swap and control SF comparing PRE and POST. The correlation between control and swap selectivity was also assessed.

The distribution of selectivity was compared between swap and control SF for each phase (two-sample Kolmogorov–Smirnov test) and equal variance was confirmed (Ansari–Bradley test of equal variance).

### Histology

After recordings were finalized several electrolytic lesions (0.1 mA, 5 s, tip negative, as described in Vermaercke et al., 2014) were made at different depths at one of the recording locations. The next day, the rats were sacrificed by injecting an overdose of sodium pentobarbital. The chest cavity was opened and a cardiac perfusion was performed with 1% paraformaldehyde in PBS, followed by 4% paraformaldehyde in PBS. The brain was removed and stored in 4% paraformaldehyde in PBS for at least 24 h.

Next, the brain was sliced in 50 μm coronal slices with a Vibratome and placed on gelatinized glass slides. A Nissl-staining was performed with 1% cresyl violet (Fluka Chemical, Sigma-Aldrich, St. Louis, MO, United States) before visually investigating the slices under the microscope. The location of the lesion was identified and compared to the atlas (Paxinos and Watson, 1982) to verify recordings were obtained in V1. For all rats the position of the lesion was confirmed to be within V1, mostly within the medial region (V1M) approximately 6 mm posterior.

## Results

Eight rats were habituated for electrophysiological recordings in awake state. To assess the effect of the exposure phase, data were collected pre- and post-exposure.

First we describe some general characteristics of how these units were sampled and selected, including a comparison of PRE and POST data on characteristics that are expected to be independent of the exposure manipulation (e.g., receptive fields, responsiveness, and baseline activity). Next, we describe the responses during the exposure phase. Finally, we turn to the central question of the manuscript: whether the exposure phase affects the independent coding of orientation and SF.

### RF Mapping

At the start of recording at each recording location the receptive field was determined to assess the presence of a visual response and correct targeting of V1, by detecting responses to flashing white squares at 23 screen positions.

**FIGURE 4:**
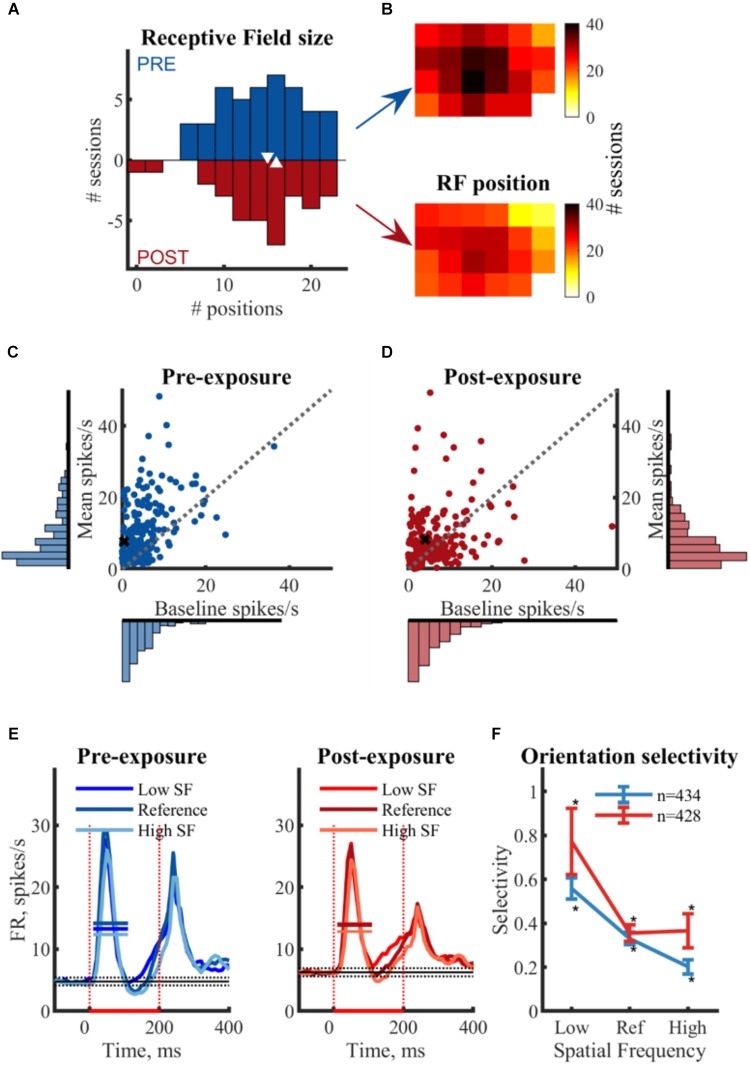
Comparison between pre- and post-exposure neural data in terms of basic functional properties. **(A,B)** Receptive field mapping of multi-unit responses. **(A)** Histogram of the RF sizes (bin size = 2) per session with median values (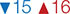). RF size is expressed as the amount of screen positions with a significant MU response (*p* < 0.05 one-sided *t*-test from BL). **(B)** Position of the MU RFs on screen. Each square represents one screen position of the flashing square. Color bar indicates the number of sessions a specific screen positions was significantly response. The bottom right corner of the screen is covered by a photocell to detect stimulus presentation and is thus not sampled during RF mapping. **(C,D)** Baseline and mean response in spikes/s: Scatterplots and associated histograms (bin size = 2 ms) represent the baseline and mean (BL corrected) responses per SU. Each dot represents the average response of one SU across all stimuli. X indicates example SUs as in Figure 3. No differences in distribution between pre- and post-exposure is observed, indicating both groups of SU’s come from a similar neural population. **(E)** Temporal response characteristics: the mean (±SEM) PSTH for all responsive and selective SUs per SF for both pre- and post-exposure. **—** mean response FR; 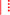 stimulus ON; 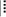 baseline response (mean ± 3^∗^SEM). **(F)** Dependence of orientation selectivity on spatial frequency: mean (±SEM) selectivity per SF for pre- and post-exposure SUs. Selectivity is calculated as the difference in normalized responses between two orientations of the same SF. Includes all SUs with significant selectivity to at least one SF, thus removing the selection bias for SUs with a strong selectivity at the reference SF. ^∗^ *t*-test from 0, *p* < 0.05.

Offline we combined all multi-unit clusters of channels for which we obtained SU data into one large multi-unit cluster per recording location. Overall we had 44 such recording locations pre-exposure, and 34 post-exposure. The screen positions yielding a significant MU response (one-sided *t*-test from BL) are considered part of the receptive field (Figure 4A,B). The median multi-unit RF size was 15 positions for PRE and 16 POST and there was no noticeable difference in the spatial distribution of the RF position between PRE and POST (Figure 4A,B). These findings indicate that the pre- and post-exposure recordings sample a similar neural population.

### Characteristics of Recorded Neurons Pre- and Post-exposure

Based on all recording locations within V1, we identified 887 and 877 putative single-unit clusters, respectively, pre- and post-exposure. Only SUs that were visually responsive and selective were used for further analysis (PRE: *n* = 239 and POST: *n* = 234). The median baseline response (-95 to 5 ms) before stimulus onset was 3.29 spikes/s PRE (min: 0 spikes/s; max: 36.37 spikes/s) and 4.18 spikes/s POST (min = 0.03 spikes/s; max: 48.83 spikes/s) (Figure 4C,D). The median stimulus response (15–115 ms, averaged across six stimuli) was 6.70 spikes/s PRE (min: 1.19 spikes/s; max: 48.26 spikes/s) and 5.82 spikes/s POST (min = -0.39 spikes/s; max: 53.01 spikes/s). Post-exposure the median baseline response is higher than pre-exposure (*z* = -3.11, *p* < 0.001) and more variable [Ansari–Bradley, *W* = 30905, *p* < 0.001 (corrected for unequal medians)], possibly due to larger noise levels. No such differences were observed for the responses. This distribution remained similar across selection criteria.

On average there were significant responses to all SFs, both pre- and post-exposure (Figure 4E). The PSTHs pre- and post-exposure showed a clear transient response with a peak around 55 ms after stimulus onset, typical for V1 (Kaliukhovich and Op de Beeck, 2018). We know from previous work (Kaliukhovich and Op de Beeck, 2018) that rat V1 neurons also show a sustained response when stimulus duration is longer, but in the current experiment the stimulus presentation was not sufficiently long to detect a sustained response (>150 ms), and the increased response after 150 ms overlaps with the off response which prevents proper identification. For that reason all our analyses are performed on the time window that captures the transient response only.

Approximately half of the responsive SUs show a significant orientation selectivity (*p* < 0.05, two-sided *t*-test as shown for the example SUs in Table 2) for at least one SF based on the difference in mean responses (PRE: 434 out of 813 SUs; POST: 428 out of 811 SUs). On a population level these selective SUs show a significant orientation selectivity to all SFs (Figure 4F, *p* < 0.05, one-sided *t*-test from 0), with decreasing selectivity for increasing SF. Again the increased variability post-exposure is noticeable. The decreasing selectivity is associated with a decreasing amount of selective clusters (Table 3) for the high SF condition. Note that a priori we tried to construct the range of SFs so that the reference SF would be close to the most optimal SF for rat V1 neurons and both other SFs would still elicit good responses (see e.g., Girman et al., 1999). Ideally we would like the same responsiveness to the low and high SF instead of the decrease in responsiveness toward the high SF. This finding has two important consequences. First, it is important that our experimental design includes a counterbalancing of control and swap SF across animals so that we have the same number of pre-exposure SUs with the low SF as swap and with the high SF as swap (as can be seen in Table 1). Second, it makes it difficult to compare the swap and control SF within a single animal to draw conclusions about effects of the exposure. Such conclusions require the pooling of data across animals.

**Table 3:** Orientation selectivity per SF.

	Low SF	Ref SF	High SF	Responsive clusters
45°	227 (29.7%)	232 (30.3%)	137 (17.9%)	765
135°	265 (30.9%)	241 (28.1%)	152 (17.7%)	859
TOTAL	492 (30.3%)	473 (29.1%)	289 (17.8%)	1624


*The amount of clusters with significant orientation selectivity per SF and per preferred orientation are shown. The responsive clusters for pre and post-exposure are combined.(%) percentage of responsive clusters with a significant preference for that SF and orientation*.

### Adaptation During Exposure Phase

**FIGURE 5:**
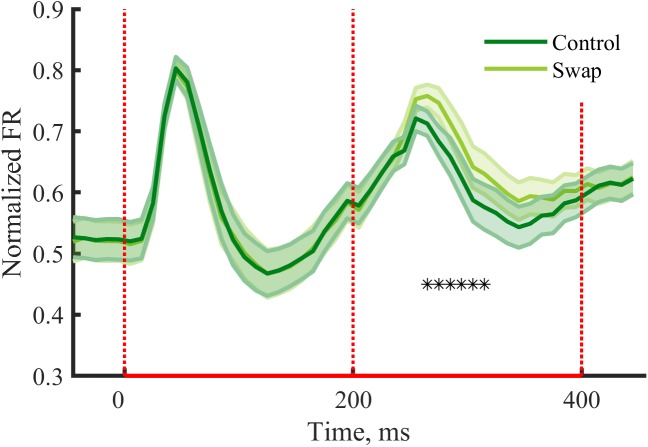
Neural responses during the exposure phase. Mean (±SEM) PSTHs of the normalized responses during the exposure phase for control and swap conditions. Normalized FR by dividing PSTH by the max peak value of the both conditions for each cluster. Response suppression is seen for the second stimulus of the pair, with a reduced suppression in the swap condition due to change in orientation on top of the change in SF. **—**^∗^*p* < 0.05, paired *t*-test for each 10 ms time bin with Bonferroni correction.

Earlier studies that manipulated temporal contiguity learning did not investigate responses during the exposure phase. Such analyses can be relevant to give insight into how neurons process the stimulus pairs. In V1 typically response suppression is observed after repeated exposure to the same stimulus (Vinken et al., 2017; Kaliukhovich and Op de Beeck, 2018), compared to a situation where one stimulus is followed by a different stimulus. We expected to see similar effects within stimulus pairs of the exposure phase, with possible differences between the conditions. For the second stimulus we observe a reduction of the peak response in both conditions as compared to the first stimulus [Swap: *t*(29) = 3.75, *p* = 0.0008; Control: *t*(29) = 6.90, *p* = 1.38^∗^10^-7^]. The suppression was stronger for the control condition than for swap condition [Figure 5, *t*(29) = -4.78, *p* = 4.73^∗^10^-5^]. The condition with a change in orientation on top of the change in SF is thus associated with a stronger release from suppression compared to the condition with only a change in SF. When looking at the response pattern more closely, we see the effect is most noticeable at the peak response and immediately after, thus from 50 to 160 ms after the onset of the second stimulus (Figure 5).

### Effect of Exposure Manipulation on Orientation Selectivity Across Spatial Frequencies

**FIGURE 6:**
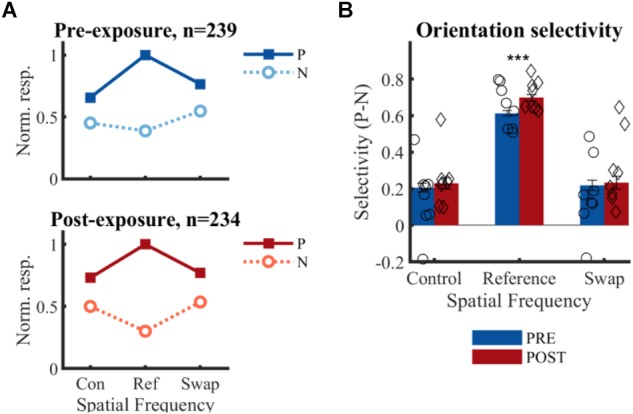
Effect of temporal contiguity on the tolerance of orientation selectivity across spatial frequencies. **(A)** Normalized responses to each orientation and SF for PRE and POST. P, preferred orientation; N, non-preferred orientation. The large selectivity for the reference SF is observed on both panels, as is the lack of change at the swap SF post-exposure as hypothesized in Figure 2E. **(B)** Mean (±SEM) selectivity of the normalized responses of all selective SUs; comparison of PRE and POST selectivity with unpaired *t*-test: ^∗∗∗^*p* < 0.001. Mean selectivity for individual animals is indicated PRE (○) and POST (♢), for individual bar plots see Figure 7.

To determine whether exposure to changes in orientation with SF has an effect on orientation preference for that swap SF, the selectivity pre- and post-exposure was determined at the swap and at the control SF. Only those clusters which showed selectivity for the reference SF (*n* = 473, Table 3) were included to compare pre- and post-exposure selectivity. Also note that the preferred orientation was determined using only the data of the reference SF (see section “Materials and Methods” for more details). The results are shown in Figure 6A. This figure looks very different than Figure 4F: the strongest selectivity is now seen for the reference SF. This is a consequence of selecting clusters and preferred orientation based on the reference SF, which artificially enhances apparent selectivity at this SF. This bias is present both pre- and post-exposure and equally affects swap and control SFs, and thus does not invalidate any conclusions drawn based on comparisons of PRE and POST, and of swap and control. These are the important comparisons for assessing whether and how the temporal contiguity manipulation, that differentiates swap from control SF, affects neural selectivity.

There is no significant change in selectivity between pre- and post-exposure at either the swap [*t*(471) = -0.33, *p* = 0.37] or control SF [*t*(471) = -0.62, *p* = 0.26] (Figure 6B). This direct comparison between PRE and POST assumes that the two recorded populations match in all respects except the exposure manipulation. The aforementioned comparisons in properties such as receptive fields suggest that this is mostly the case, but it is difficult to guarantee this beyond any doubt. For that reason, we also compare the swap and control condition within the same sample of neurons in the POST data. The selectivity in the swap and control SF did not differ significantly from each other in the POST data [*t*(233) = 0.07, *p* = 0.95]. For completeness, we performed the same comparison in the PRE data, which also did not reveal any difference [*t*(238) = 0.38, *p* = 0.70]. In addition, the selectivity in the swap [PRE: *t*(238) = 7.79, *p* = 0.21^∗^10^-12^; POST: *t*(233) = 6.47, *p* = 0.58^∗^10^-9^] and control [PRE: *t*(238) = 8.28, *p* = 0.09^∗^10^-13^; POST: *t*(233) = 7.61, *p* = 0.07^∗^10^-11^] SF differed significantly from the reference condition, which is not surprising given several selection biases in our experiment (reference SF is the most optimal SF in the literature; decisions about selective units and preferred orientation were based on reference SF). There was also a significant increase in selectivity from pre- to post-exposure for the reference SF [*t*(471) = 3.76, *p* = 0.0001], which might be in line with previously reported effects of stimulus exposure (Frenkel et al., 2006).

**FIGURE 7:**
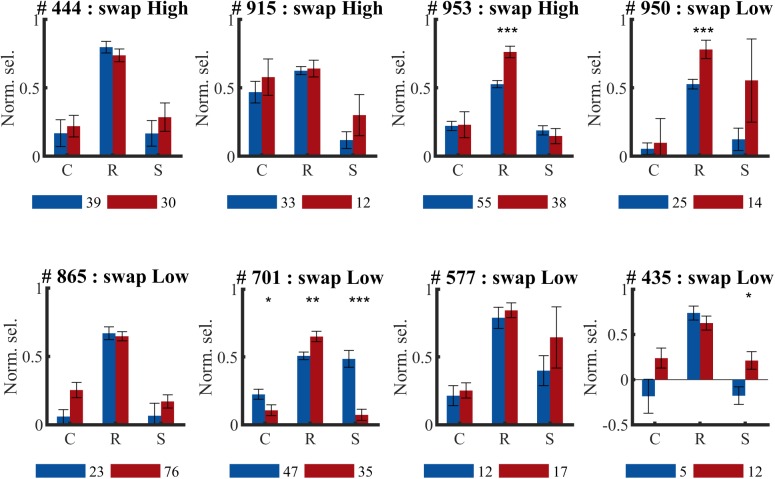
Effect of temporal contiguity on the tolerance of orientation selectivity across spatial frequencies per animal. Mean (±SEM) orientation selectivity per SF and per phase (PRE and POST) of all selective SUs per animal (# animal ID) (same data as in Figure 6, now per animal). C, control SF; R, reference SF; S, swap SF. Number below each graph indicate amount of SUs collected during pre- (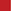) and post-exposure (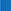) sessions. Comparison of PRE and POST selectivity with unpaired *t*-test: ^∗^*p* < 0.05, ^∗∗^*p* < 0.01, ^∗∗∗^*p* < 0.001.

For three animals the swap condition involved the high SF, for the other animals the low SF. The number of animals and neurons in these two conditions was not the same due to the variability in the amount of SUs that could be collected in each session and between animals (see Table 1). Also at the level of individual animals, we did not see any indication of the predicted effect, as can be checked in detail in Figure 7. This effect could be visible as a reduction of selectivity in the post-exposure swap condition compared to pre-exposure swap condition. In this case we compare responses to the same stimuli, so any differences are not contaminated by possible differences between high and low SF. There is no overall reduction, one animal seems to show such an effect (#701; red lower than blue in condition S in Figure 7), but other animals show a trend in the opposite direction. The predicted effect could also be visible as a reduction of selectivity in the post-exposure swap condition (red in condition S) compared to the post-exposure control condition (red in condition C) in the same animal or the same group of animals (group = which SF is assigned to swap). In this case we do a paired-sample comparison (data from the same neurons), but the data are collected with different stimuli. Again we see variation among animals, and no clear tendency in the predicted direction.

**FIGURE 8:**
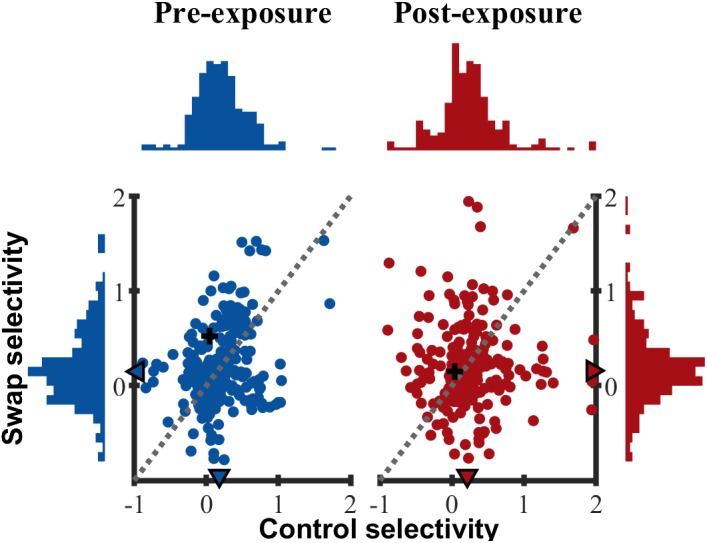
Selectivity distribution. Scatterplot of control versus swap selectivity based on normalized responses for PRE and POST. Histograms indicate the distribution of selectivity for control (horizontal) and swap (vertical) SF. ▲ median values for all animals. + indicates example SUs shown in Figure 3.

Up to now we only tested for differences in the mean selectivity between swap and control with parametric tests. In addition, we also verified that the distribution of selectivity (Figure 8) in the swap and control SF were similar, both pre- (two-sample Kolmogorov–Smirnov test, *p* = 0.57) and post-exposure (*p* = 0.20). All distributions were right-tailed, equal in shape and variability (Ansari–Bradley test of equal variance, PRE *p* = 0.14; POST *p* = 0.87) with slightly positive medians. As shown in the scatter plots of Figure 8 there was a weak positive correlation between control and swap selectivity pre-exposure (*R*_spearman_ = 0.205, *p* = 0.002), but not post-exposure (*R*_spearman_ = 0.031, *p* = 0.64).

We report a null result, finding no difference between the swap and the control SF after exposure training. The power of an experiment is an important determinant for how we can interpret such a null result. To compute this power, we assumed that we are after a reduction of selectivity in the swap condition of 50% of the selectivity in the control condition, which is similar to the effect observed by Li and DiCarlo (2010) who used a similar number of exposure trials. Note that here we only count the number of trials in a single exposure phase, which is an underestimation of the total exposure in our design that combines an accumulation of exposure effects within as well as between sessions. A reduction of selectivity of 50% from the measured normalized selectivity of 0.22 in the pre-exposure swap condition would amount to a reduction of 0.11 in normalized selectivity. The selectivity can be summarized by a simple number per neural site, with different sites pre and post exposure, thus what we need to assess is the power of a regular two-sample *t*-test (the test statistic that we mostly used here). With a standard deviation across neurons of 0.43 this amounts to an effect size of 0.11/0.43 = 0.26. With these parameters and a sample size above 230 we computed that we have at least 80% probability to reject the null hypothesis when we test with a two-sided test (power of 0.8). This is conventionally taken as a sufficiently powerful experiment. In this case where we have an explicit prediction about the direction of the effect, it would also be valid to go for a one-sided test in which case the power would increase to 0.90. Given these high values we have high confidence that our null result is not a false negative.

## Discussion

According to the temporal contiguity hypothesis, a repeated exposure to a change in orientation across temporally contiguous changes in SF, would reduce the orientation selectivity for that SF, but not for other SFs. Contrary to earlier results in primates in the context of high-level object selectivity, we did not observe these effects in V1 of rats. Neither the selectivity of the swap nor of the control condition changed after exposure. The absence of effect was found in direct comparisons of a swap and control condition, and in addition in a comparison of pre-exposure to post-exposure recordings sampling from similar neural populations. The response pattern during the exposure phase suggest that the neurons were sensitive to the difference between swap and control conditions during exposure.

The lack of an altered selectivity after exposure is not in line with previous evidence in primates (Wallis and Rolls, 1997; Li and DiCarlo, 2008, 2010; Van Meel and Op de Beeck, 2018). This could be due to several differences between the current study and the electrophysiology experiments done by Li and DiCarlo (2008, 2010). The intended difference is that we target a different level of the visual processing hierarchy and a set of dimensions coded at that level that are often assumed to be the basic building blocks of all further processing. If the stability of these building blocks would be important, then it might make sense that they are not easily changed by exposure manipulations. Earlier protocols that induced plasticity in V1 (O’Hashi et al., 2007; Yoshida et al., 2012), presented more invasive changes in the visual experience than the current exposure protocol with changing pairs of stimuli, which was modeled after the protocol for influencing size invariance through temporal contiguity exposure in monkey ITC (Li and DiCarlo, 2010). Yao and Dan (2001) also made use of pairs of differently oriented stimuli to induce plasticity, but the exposure effects were determined within neurons and the stimuli were optimized for that individual neuron. Additionally the effect also disappeared a few minutes after exposure and the observed shift was much less than the 90° orientation difference we used. Higher visual cortex, like ITC, constantly has to adapt to new environments and keep learning well into adulthood (Kourtzi and DiCarlo, 2006), this is less critical for the lower visual cortex. Even though we did not observe effects of temporal contiguity exposure in V1 with our design, it is possible that effects would occur in V1 with longer manipulations, with manipulations that fall earlier in development, or with manipulations that target other examples of invariance (such as the phase invariance of complex cells).

In addition to this intended difference with earlier work on temporal contiguity, we also studied a different species, rats instead of monkeys. We cannot exclude the possibility that the effect can be found in primate V1, but not in rodent. However, we consider this unlikely. Even though they differ in anatomical structure, both cortices show great similarities on a functional level (Van den Bergh et al., 2010; Kaschube, 2014). Both species, as well as all other placental mammals that have been studied (Scholl et al., 2017), show orientation selectivity, a clear retinotopic map, sensitivity to contrast changes, SF tuning, etc. Finally, finding the effect in rodents would have offered some additional advantages. It has been suggested that smaller brains may actually be preferable to study basic mechanisms due to their reduced complexity (Zoccolan et al., 2015). Additionally, rodents have been the preferred species in recent studies of the underlying neural computations of orientation preference (Varela et al., 1997; Felsen et al., 2002; Ghodrati et al., 2016).

There are several other differences between our experiment and the earlier work of Li and DiCarlo, but these differences are inherently related to the features represented in ITC compared to area V1. Monkey IT neurons are tuned in a high-dimensional feature space. In contrast, in V1 the tuning is typically described in terms of a small set of dimensions, such as orientation, SF, and direction of motion. This has important consequences on the possibilities to design independent exposure phases that show no interference between sessions. In ITC, each exposure phase includes a different pair of complex objects, and it is assumed that the temporal contiguity manipulation for one pair on one session does not interfere with a next session, as shown by the unchanged selectivity of an unexposed control object (Li and DiCarlo, 2008). Said otherwise, even after many previous exposure phases in a monkey it is assumed that neurons are still naive as if there would not have been any previous exposures, at least as long as a new recording session and exposure involves different objects than previous days. In V1 we cannot make that assumption. The number of different stimuli is much smaller due to the smaller feature space that is represented, which will result in interference between temporal contiguity manipulations of different days. One solution could have been to restrict exposure and test stimuli to small parts of the visual field, but even then it would be difficult to fully avoid interference. In addition, we only performed single-unit discrimination offline, and the online receptive fields and online selectivity would not be sufficient to make a proper choice of exposure stimuli and locations for each single unit. For these reasons we decided to stick to one specific exposure protocol per animal and counterbalance the swap and control conditions across animals. The exposure protocol involves full-screen stimuli so that all relevant visual field positions are exposed to these stimuli.

In terms of effect size, we think our design with repeating exposures across days should make effects larger rather than smaller, because exposure effects could accumulate across days. However, as a disadvantage, our design does not allow us to assess exposure effects in individual units as we do not have both a PRE and a POST measurement for each unit. For this reason we rely upon population-level statistics to assess the effect of the temporal contiguity manipulations. Our dataset includes a relatively high number of neurons so that we have the power to also interpret null results. Thus, if an effect of a similar size as in the studies of Li and DiCarlo (2008, 2010) existed, we should have been able to detect it. After only 400 swap trials, they observed approximately 50% change in the normalized SU response of monkey ITC (Li and DiCarlo, 2008). Using MU activity, they observed an even larger effect (a reversal in preference) after 800 trials. Considering that this comparison ignores the potential for the accumulation of effects across exposure sessions in our design, this effect size would be a conservative assumption. Our power with this conservative estimate of the expected effect size is 0.80–0.90. We are thus confident that if an effect were present in rat V1, our dataset would allow us to detect it. The fact that we observe a null result in this design, and not even a trend in the direction of an effect, makes us confident that with this design there is no effect of temporal contiguity manipulations on the degree to which orientation selectivity is influenced by variations in SF.

## Conclusion

Although some strong evidence has been given for the temporal contiguity hypothesis, little is known about the extent of the effect, and whether it can generalize to other brain area’s and stimulus features. We showed that temporal contiguity learning is unlikely to alter the orientation selectivity in V1. Further research is needed to define whether temporal contiguity learning could also play a role in other brain areas or properties.

## Data Availability

The datasets analyzed for this study can be found in the Open Science Framework (https://osf.io/a8ejt).

## Ethics Statement

The protocol was approved by the Ethical Committee for Animal Experiments at the KU Leuven (P119/2014).

## Author Contributions

EC, DK, and HOdB designed the research. EC and LV performed the research and analyzed the data. EC and HOdB wrote the manuscript.

## Conflict of Interest Statement

The authors declare that the research was conducted in the absence of any commercial or financial relationships that could be construed as a potential conflict of interest.
